# Author Correction: Constraining Earth’s core composition from inner core nucleation

**DOI:** 10.1038/s41467-025-66094-z

**Published:** 2025-11-24

**Authors:** Alfred J. Wilson, Christopher J. Davies, Andrew M. Walker, Dario Alfè

**Affiliations:** 1 School of Earth and Environment, University of Leeds, Leeds, UK; 2 Department of Earth Sciences, University of Oxford, Oxford, UK; 3 Department of Earth Sciences, University College London, London, UK; 4 London Centre for Nanotechnology, University College London, London, WC1H 0AH UK; 5 Dipartimento di Fisica “Ettore Pancini”, Universita‘ di Napoli “Federico II”m, Napoli, Italy

**Keywords:** Geophysics, Core processes, Core processes, Mineralogy

Correction to: *Nature Communications* 10.1038/s41467-025-62841-4, published online 04 September 2025

In the version of the article initially published, equation 7 was erroneous and should not have been present. This equation was not used in the study and did not contribute to any other equations of the article. The original equation 7 has been deleted and all subsequent equations have now been renumbered in the HTML and PDF versions of the article. The Supplementary information accompanying this amendment presents the original, uncorrected equation 7 for comparison. This change does not influence the result, discussion or implications of the article.

## Supplementary information

Original, uncorrected Eq. (7)

