# Supplementary material for: Constraining Earth’s core composition from inner core nucleation
Source: Nat Commun. 2025 Sep 4;16:7685. doi: 10.1038/s41467-025-62841-4 (PMC12411617; doi:10.1038/s41467-025-62841-4)
Supplement: Supplementary file 1 — Supplementary Information [file 41467_2025_62841_MOESM1_ESM.pdf]

Supplementary Information: Constraining Earth's core composition from inner core nucleation

Alfred J. Wilson[1*], Christopher J. Davies[1], Andrew M. Walker[2], Dario Alfè[3,4,5]

[1*]School of Earth and Environment, University of Leeds, Leeds, LS2 9JT, United Kingdom.
[2]Department of Earth Sciences, Organization, Oxford, OX1 2JD, United Kingdom.
[3]Department of Earth Sciences, University College London, London, WC1E 6BT, United Kingdom.
[4]London Centre for Nanotechnology, University College London, Thomas Young Centre, London, WC1H 0AH, United Kingdom.
[5]Dipartimento di Fisica "Ettore Pancini", Universita' di Napoli "Federico II"m, Napoli, 80126, Italy.

*Corresponding author(s). E-mail(s): a.j.wilson1@leeds.ac.uk;

This document contains supplementary information for the article "Constraining Earth's core composition from inner core nucleation", which uses molecular dynamic simulations of supercooled Fe-C liquids to assess the conditions required to nucleate the Earth's solid inner core from these liquids.

Waiting time for inner core nucleation

In this study we use molecular dynamic simulations to estimate the nucleation rate (Eq. 9) of supercooled Fe-C liquids. The aim is to evaluate the rate at which the same liquids would nucleate solid particles if present in the Earth's core. The inverse of nucleation rate is the waiting time (Eq. 10) before a nucleation event will be observed. By examining the relationship between supercooling and the waiting time to observe the nucleation of a solid particle which is large enough to escape remelting, we can

understand the supercooling which would be necessary to nucleate the inner core from these liquids. To find the minimum supercooling required for such a nucleation event, we must know the maximum waiting time τ_w^{Earth} for which the Earth's liquid core might have been supercooled, where this waiting time has units of time volume ($\tau_w^{Earth} = t_{supercooled} \times v_{supercooled}$). A maximum supercooling can be estimated by assuming that the inner core froze in the recent past and its entire volume was supercooled liquid for the preceding 1 Gyrs. This would require that the core has been cooling at $\sim$ 400 K Gyrs^{-1} if 400 K of supercooling was needed for inner core nucleation, significantly higher than previous estimates [1]. The corresponding waiting time for this case is $2.4{\times}10^{35}$ s m^3. A less extreme case would involve a smaller volume of the core being supercooled, implying that not all of the inner core froze in the immediate past. If the supercooled region of the core were half of the present day inner core radius, the available waiting time would be 3.1 $\times 10^{34}$ s m^3, again assuming supercooling for 1 Gyrs. For a more geophysically plausible case we consider an inner core age of 300 Myrs [1] and a growth history from our previous study [2] (yellow line of figure 1b). This would imply that the inner 639 km radius of the core was supercooled and froze rapidly upon inner core nucleation and that the upper 582 km of the inner core grew slowly over the following 300 Myrs. In this case, if the central volume of the core was supercooled for 1 Gyrs, the corresponding availible waiting time was 3.5 s m^3. Ultimately, because the waiting time scales exponentially with supercooling (Eq. 10), the difference between extreme and plausible cases is small in terms of supercooling required to achieve them.

Whilst, all of these cases still involve rapidly freezing large volumes of the inner core, it is not obvious what observable signature these might have. Seismologically, the innermost inner core could be the result of distinct crystal textures formed through rapid freezing [3]. Similarly, if the outermost $\sim$ 100 km thick anisotropic layer of the inner core could have grown slowly and all material beneath this might have been frozen rapidly from supercooled liquids. Dynamically, the rapid growth of the inner core from supercooled liquids could occur on a timescale shorter than 10,000 years [4, 5], with anomalous outer core dynamics lasting for a similar length of time [3]. The sparsity of the palaeomagnetic record over past 1 Gyrs [6] means that a magnetic signature of inner core nucleation may not be preserved. Therefore, a requirement for rapid growth to be captured in the palaeomagnetic record has yet to be defined.

Geophysically compatible supercooling of Earth's liquid core

Attempts to calculate the supercooling required to nucleate the Earth's inner core from pure Fe find that the liquids must be cooled to at least 500 K below their melting point [2, 7–9], even using the maximum waiting time of $2.4{\times}10^{35}$s m^3, discussed above. To evaluate whether this degree of supercooling could have existed in the deep Earth several geophysical observations can be applied.

The location of the inner core boundary represents a phase transition in the Earth's core from solid to liquid. If the inner core boundary is in thermodynamic equilibrium, then this location is where the temperature of the core is equal to its melting

temperature and at all locations deeper than this material is cooler than the melting temperature. The maximum supercooling of the core which can remain consistent with the seismically observed size of the present day inner core is therefore defined by the separation of core temperature profile and melting temperature profile at the centre of the Earth under the constraint that these profiles intersect at the inner core boundary. In our previous study [5], we explored a range of melting curves and temperature profiles to find that $\sim$420 K is that largest supercooling which can still explain the observed inner core radius. Although, this requires that the region now occupied by the inner core was stratified and isothermal prior to nucleation.

Other constraints on the maximum supercooling of the core come from the thermal history of the deep Earth and the palaeomagnetic record. Our previous study [3] reviews all available constraints to conclude that the persistent generation of the Earth's geomagnetic field over the past 3.5 Gyrs and the present-day structure of the core permit a supercooling of less than 100 K.

Fe-C Embedded Atom Model

The embedded atom model (EAM) of this study underpins the molecular dynamics simulations performed. The model is an extension of that developed in out previous work [5] which adopts the Fe contributions of Alfè et al. [10]. Fitting of the parameters of this model follow the approach of Wilson et al.[5] where the energies, pressure and configurations of ab initio molecular dynamic (AIMD) calculations are used as training data. The absolute energy difference between classical molecular dynamics using this EAM and ab initio molecular dynamics (not included in the training dataset) is less than 0.15 eV per cell (figure 1). The absolute pressure difference for the same comparison is always less than 60 MPa (figure 2).

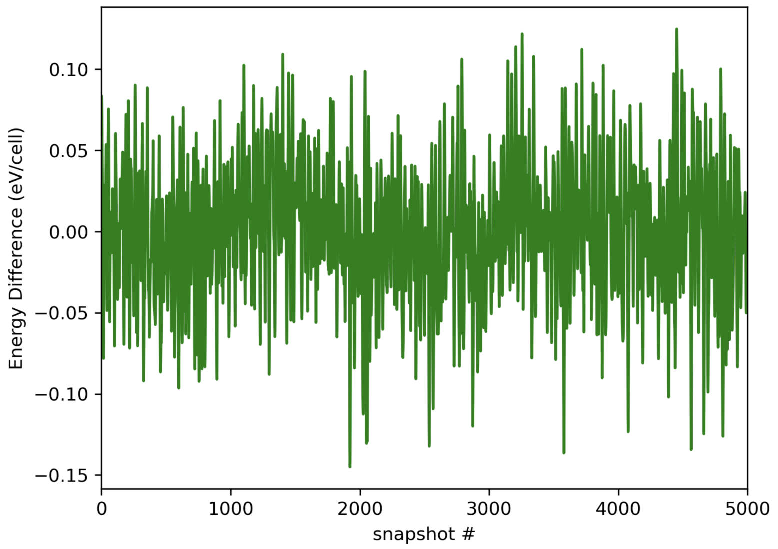

Fig. 1 Energy differences between classical and ab initio molecular dynamic simulations (run using LAMMPS and VASP, respectively) of $Fe_{115}C_{13}$ liquid at 5000 K (in the NVT ensemble) with a volume of 914.94 Å^3. Snapshots are sequential and each is separated by 100 fs.

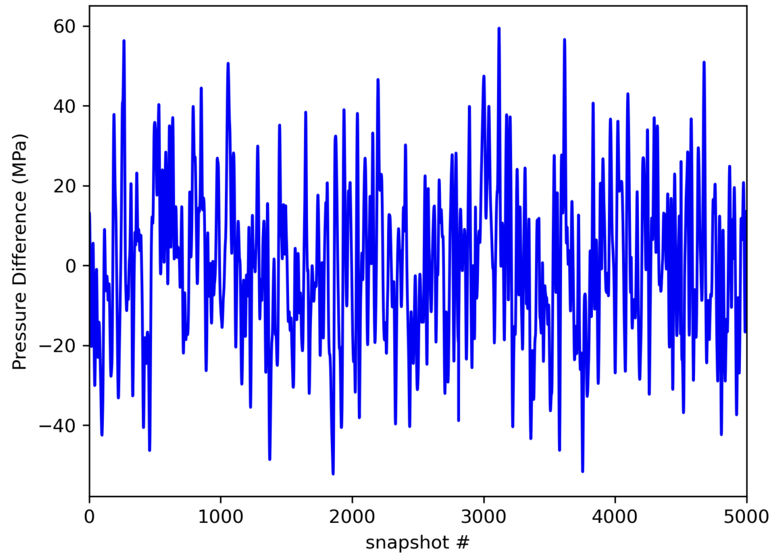

Fig. 2 Pressure differences between classical and ab initio molecular dynamic simulations (run using LAMMPS and VASP, respectively) of $Fe_{115}C_{13}$ liquid at 5000 K (in the NVT ensemble) with a volume of 914.94 Å^3. Snapshots are sequential and each is separated by 100 fs.

Pair distribution functions are used to compare the liquid structure classical molecular dynamic and ab initio molecular dynamics simulations of $Fe_{0.9}C_{0.1}$ liquids. These comparisons reveal that the structure of AIMD simulations is accurately reproduced up to 6 Å separation. Deviations at larger separations can be attributed to finite system sizes, especially for ab initio calculations where 128 atoms are simulated. The pair distribution functions for Fe-Fe, Fe-C and C-C are shown in figures 3-5 where C-C interactions are least complete because of the rarity of interactions (due to the relatively low concentration of C in these simulations). The difference between the C-C distribution for CMD and AIMD is due to the difference in system sizes (64,000 atoms and 128 atoms, respectively.

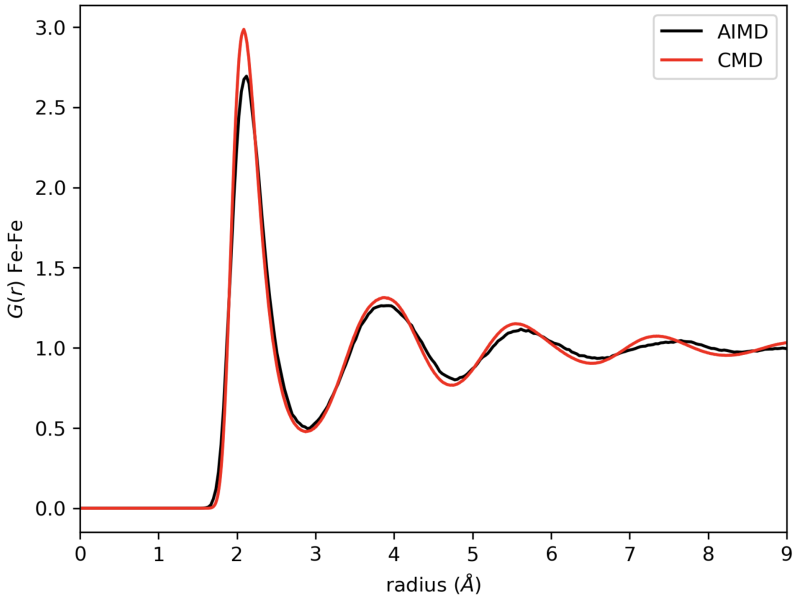

Fig. 3 Pair distribution function for Fe-Fe interactions in an ab initio molecular dynamic simulation (black) and a classical molecular dynamic simulation (red) using the embedded atom model of this study. Both simulations are of $Fe_{0.9}C_{0.1}$ at 360 GPa and 5000 K in the NVT ensemble.

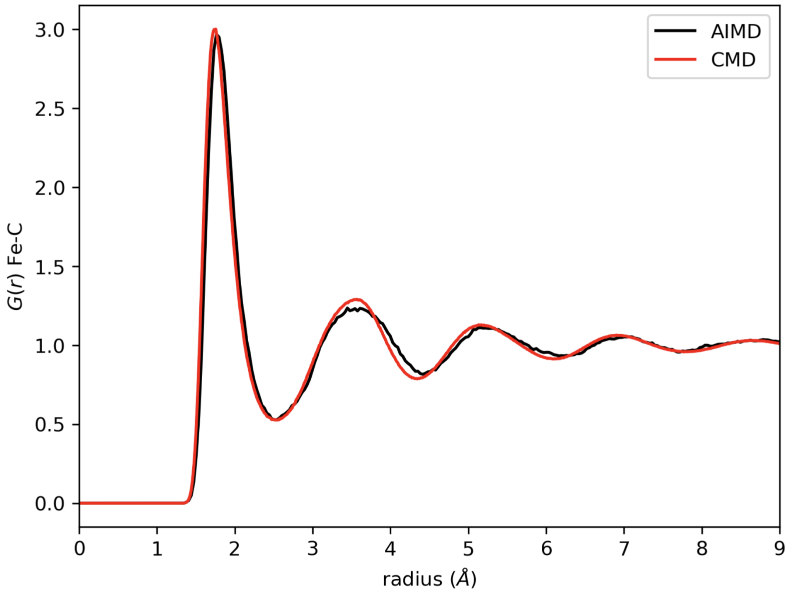

Fig. 4 Pair distribution function for Fe-C interactions in an ab initio molecular dynamic simulation (black) and a classical molecular dynamic simulation (red) using the embedded atom model of this study. Both simulations are of $Fe_{0.9}C_{0.1}$ at 360 GPa and 5000 K in the NVT ensemble.

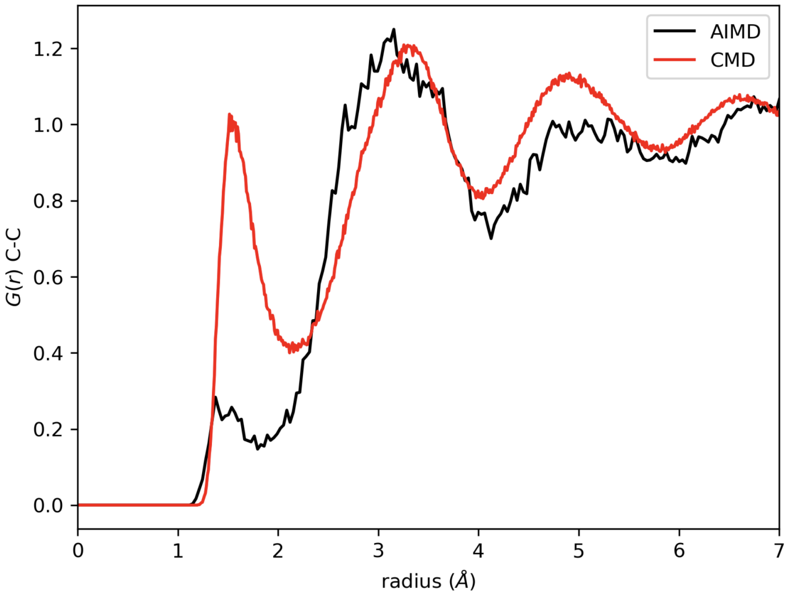

Fig. 5 Pair distribution function for C-C interactions in an ab initio molecular dynamic simulation (black) and a classical molecular dynamic simulation (red) using the embedded atom model of this study. Both simulations are of $Fe_{0.9}C_{0.1}$ at 360 GPa and 5000 K in the NVT ensemble.

References

[1] Davies, C.: Cooling history of earth's core with high thermal conductivity. Physics of the Earth and Planetary Interiors **247**, 65–79 (2015)

[2] Wilson, A.J., Walker, A.M., Alfè, D., Davies, C.J.: Probing the nucleation of iron in earth's core using molecular dynamics simulations of supercooled liquids. Physical Review B **103**(21), 214113 (2021)

[3] Wilson, A., Davies, C., Walker, A., Alfe, D., Pozzo, M., Deuss, A.: Accepted: The formation and evolution of earth's inner core. Nature Reviews Earth & Environment **TBC**(TBC), (2025)

[4] Sun, G., Xu, J., Harrowell, P.: The mechanism of the ultrafast crystal growth of pure metals from their melts. Nature materials **17**(10), 881–886 (2018)

[5] Wilson, A.J., Alfè, D., Walker, A.M., Davies, C.J.: Can homogeneous nucleation resolve the inner core nucleation paradox? Earth and Planetary Science Letters **614**, 118176 (2023)

[6] Bono, R.K., Paterson, G.A., Boon, A., Engbers, Y.A., Michael Grappone, J., Handford, B., Hawkins, L.M., Lloyd, S.J., Sprain, C.J., Thallner, D., *et al.*: The pint database: a definitive compilation of absolute palaeomagnetic intensity determinations since 4 billion years ago. Geophysical Journal International **229**(1), 522–545 (2022)

[7] Huguet, L., Van Orman, J.A., Hauck II, S.A., Willard, M.A.: Earth's inner core nucleation paradox. Earth and Planetary Science Letters **487**, 9–20 (2018)

[8] Davies, C., Pozzo, M., Alfè, D.: Assessing the inner core nucleation paradox with atomic-scale simulations. Earth and Planetary Science Letters **507**, 1–9 (2019)

[9] Sun, Y., Zhang, F., Mendelev, M.I., Wentzcovitch, R.M., Ho, K.-M.: Two-step nucleation of the earth's inner core. Proceedings of the National Academy of Sciences **119**(2), 2113059119 (2022)

[10] Alfè, D., Gillan, M., Price, G.: Complementary approaches to the ab initio calculation of melting properties. The Journal of chemical physics **116**(14), 6170–6177 (2002)
