# Supplementary material for: Author Correction: Constraining Earth’s core composition from inner core nucleation
Source: Nat Commun. 2025 Nov 24;16:10318. doi: 10.1038/s41467-025-66094-z (PMC12644654; doi:10.1038/s41467-025-66094-z)
Supplement: Supplementary file 1 — Original, uncorrected Eq. (7) [file 41467_2025_66094_MOESM1_ESM.pdf]

Inner core nucleation subject to a supercooling of 200–400 K has potentially significant implications for interpreting the structure, dynamics, and evolution of Earth's core. The predicted supercooling would delay the inner core formation age predicted by core evolution models by $O(100)$ Myrs[54,55]. In classical evolution models with high core conductivity[56] this delay would likely imply a lack of power available to the dynamo prior to inner core formation, in conflict with paleomagnetic observations[57,58]. This observation lends support to evolutionary scenarios that include long-lived dynamo power supplied by precipitation of oxides at the CMB[59–63], though the effect of C on the partitioning behaviour at the CMB has not been systematically evaluated and may influence the power provided by precipitation. Sudden rapid growth of the inner core following nucleation may leave a signature in the paleomagnetic record owing to the additional latent heat and gravitational power to the dynamo[29], though the expected influence on field intensity and variability has not yet been studied in detail. Finally, delayed inner core formation may influence texturing of the inner core, for example by trapping liquids in the solid[54], and has been correlated with the existence of the innermost inner core[55].

Methods

Interatomic potential

We use CMD simulations of liquid $Fe_{1-x}C_x$ to characterise nucleation behaviour at a range of T and x^C. To describe the interatomic forces and system energies in our simulations, we develop an EAM that is trained on ab initio calculations. The model is fit to reproduce the positions, energy (E) and P of snapshots from ab initio molecular dynamics (AIMD) calculations run using the VASP software package[64] with the projector augmented wave method[65] and the PW91 generalised gradient approximation functional[66]. Details of these calculations follow Wilson et al.[33], which shares some of the same AIMD data at low x^C used for fitting the potential. The EAM potential is validated against a separate suite of AIMD snapshots to ensure that E and P are accurately reproduced. The root mean square of fluctuations in E is determined to be 0.292 and 0.316 eV per cell at 5000 K for $Fe_{0.95}C_{0.05}$ and $Fe_{0.9}C_{0.1}$, respectively, far less than k_B/T (0.431 eV). Reproduction of liquid structure is confirmed by comparison of radial distribution functions, where average positions of neighbouring atoms in CMD simulations are within 0.05 Å of AIMD simulations for all interactions and all volume V, T, x^C conditions. Further comparison of this potential with AIMD validation data is provided in the Supplementary Information.

AIMD simulations are performed by melting systems of 128 atoms with different carbon fractions (close to 20, 10 and 5 mol%) at 10,000 K for 1 ps before equilibrating at a target T (4000, 5000 and 6000 K) for 1 ps and evolving the system at the target T for 30 ps. The simulation cell volume is tuned for each composition and target T to achieve a P of 360 GPa. From the final 30 ps of simulation time, configurations are selected at every 100 fs as data on which the EAM is trained. The total energy E of a $Fe_{1-x}C_x$ system is defined by the EAM as the sum of contributions from all atomic interactions

$$E = \sum_{i_{Fe}} E_i^{Fe} + \sum_{i_C} E_i^C + \sum_{i_{FeC}} E_i^{FeC}. \tag{1}$$

Each interaction between atoms i and j contains repulsive Q and embedded F contributions. Q depends on the interatomic distance r_{ij}, which also defines an electron density ρ_{ij} on which F depends. E for each type of interaction is given by

$$\begin{aligned} E_i^{Fe} &= Q_i^{Fe} + F^{Fe}(\rho_i^{Fe}) \\ &= \sum_{i<j} \epsilon^{Fe}\left(a^{Fe}/r_{i_{Fe}j_{Fe}}\right)^{n^{Fe}} - \epsilon^{Fe}\dot{C}^{Fe}\sqrt{\rho_i^{Fe}}, \end{aligned} \tag{2}$$

$$\begin{aligned} E_i^{C} &= Q_i^{C} + F^{C}(\rho_i^{C}) \\ &= \sum_{i<j} \epsilon^{C}\left(a^{C}/r_{i_{C}j_{C}}\right)^{n^{C}} - \epsilon^{C}\dot{C}^{C}\sqrt{\rho_i^{C}}, \end{aligned} \tag{3}$$

$$\begin{aligned} E_i^{FeC} &= Q_i^{FeC} \\ &= \frac{1}{2}\sum_{i_{Fe}\neq j_C} \epsilon^{FeC}\left(a^{FeC}/r_{i_{Fe}j_C}\right)^{n^{FeC}}, \end{aligned} \tag{4}$$

where the respective densities are

$$\rho_i^{Fe} = \sum_{j_{Fe}\neq i_{Fe}} \left(a^{Fe}/r_{i_{Fe}j_{Fe}}\right)^{m^{Fe}} + \sum_{j_C} \left(a^{FeC}/r_{i_{Fe}j_C}\right)^{m_{FeC}}, \tag{5}$$

$$\rho_i^{C} = \sum_{j_C\neq i_C} \left(a^{C}/r_{i_C j_C}\right)^{m^C} + \sum_{j_{Fe}} \left(a^{FeC}/r_{i_C j_{Fe}}\right)^{m_{FeC}}, \tag{6}$$

and

$$\rho_i^{FeC} = \sum_{j=1, j\neq i}^{N_C} \left(a^{FeC}/r_{ij}\right)^{m^{FeC}}. \tag{7}$$

Here, ϵ, a, n, m and $\dot{C}$ are free parameters to be fit for each interaction and are reported in Table 2. The primary difference to the parameters found in our previous study[33] is a reduction in ϵ^{FeC} and ϵ^C, as well as a^{FeC} and a^C.

Melting temperatures

The melting temperatures of $Fe_{1-x}C_x$ are calculated with coexistence simulations using the EAM potential and the LAMMPS simulation package[67]. Systems of 128000 atoms are arranged into a long periodic cell where the x-axis is 3 times the length of the y and z axes. All atoms are initially arranged in a hexagonally close-packed structure with C atoms randomly replacing Fe atoms to achieve the desired concentration. This substitutional model is chosen based on ab initio evidence that C substitutions produce a lower free-energy state than interstitial C[47]. The positions of atoms in the central 50% of the simulation are initially fixed in space, whilst the other half is melted at 10,000 K for 5 ps. This procedure establishes the two-phase system. The entire system is then evolved at a target T under the NVT ensemble, where the number of atoms, volume and temperature are held constant, for 1 ps to establish the target average kinetic energy. Finally, the system is evolved for 10 ps under the NVE ensemble (constant Number of atoms, Volume of the system and Energy of the system), allowing the solid region of the system to grow or melt. This process is repeated a minimum of 50 times for each composition, temperature and volume initial condition (where the volume is chosen such that the pressure of the system is 360(±2) GPa at the conditions of interest). As a result, a wide variety of configurations are sampled.

Once a system has reached equilibrium, the T will lie on the melting curve, meaning that the time-averaged T and P provide a single T_m. The random distribution of C in the initial system provides many different initial x^C for the solid and freezing and melting of the solid allow for C partitioning between the solid and the liquid. Systems with $x^C > 0.05$ in the solid see much of the solid melt before freezing a lower x^C solid. This process shows that whilst C cannot diffuse freely in the solid over the timescale of these simulations, systems tend towards chemical equilibrium through freezing and melting. We note that we do not observe evidence of superionicity in these simulations, in line with previous ab initio studies[47], although this behaviour has been reported elsewhere, C-bearing Fe alloys at core conditions[68].
